# Effect of Graphene Oxide on the Properties of Polymer Inclusion Membranes for Gold Extraction from Acidic Solution

**DOI:** 10.3390/membranes12100996

**Published:** 2022-10-14

**Authors:** Siti Madiha Husna, Abdul Hafidz Yusoff, Mythili Mohan, Nur Aina Azmi, Teo Pao Ter, Noor Fazliani Shoparwe, Ahmad Ziad Sulaiman

**Affiliations:** 1 Gold Rare Earth and Material Technopreneurship Centre (GREAT), Faculty of Bioengineering and Technology, Universiti Malaysia Kelantan, Kelantan, Jeli 17600, Kelantan, Malaysia; 2 Benua Sunda Cari Gali Sdn Bhd.No 6, Medan Pusat Bandar 1, Seksyen 9, Bandar Baru Bangi 43650, Selangor, Malaysia; 3 Advanced Material Research Cluster, Faculty of Bioengineering and Technology, Universiti Malaysia Kelantan, Kelantan, Jeli 17600, Kelantan, Malaysia

**Keywords:** polymer inclusion membrane (PIM), graphene oxide (GO), membrane characterization, gold extraction

## Abstract

The cyanidation leaching method is hazardous to the environment, but it is widely applied in the gold mining process because it is effective for gold extraction. This study fabricates polymer inclusion membranes (PIMs), which have environment-friendly properties, with graphene oxide (GO) as an alternative to the cyanidation leaching method for gold extraction. Poly(vinylidenefluoride-co-hexa-fluoropropylene)-based PIMs with different GO concentrations in five membranes (i.e., M1 (0 wt.%), M2 (0.5 wt.%), M3 (1.0 wt.%), M4 (1.5 wt.%), and M5 (2.0 wt.%)) are studied for their potential to extract gold from a hydrochloric acid solution. The membranes are prepared using di-(2-ethylhexyl) phosphoric acid as the extractant and dioctyl phthalate as the plasticizer. Scanning electron microscopy, Fourier-transform infrared spectroscopy, thermogravimetric analysis, ion exchange capacity, and water uptake are used to characterize the physical and chemical properties of the fabricated PIMs. The results show that the optimized membrane for gold extraction is M4 (1.5 wt.% GO), which yields a better performance on thermal stability, ion exchange capacity (IEC), and water uptake. M4 (1.5 wt.% GO) also exhibits a smooth and dense structure, with the maximum extraction efficiency obtained at 84.71% of extracted gold. In conclusion, PIMs can be used as an alternative for extracting gold with a better performance by the presence of 1.5 wt.% GO in membrane composition.

## 1. Introduction

The current wide application of gold metal has increased the demand for gold production [1]. In industrial processes, gold is obtained via a hydrometallurgical technique, called cyanidation leaching. This method involves the conversion of gold ores to a gold solution commonly in the form of a cyanide complex solution before the extraction. Unfortunately, toxic HCN gas from CN^−^ ions is freely released during cyanidation leaching; hence, this method is not environment-friendly in terms of the human body health [1]. In addition, a large amount of thiocyanate is discharged into the tailing dams during the process. Thiocyanate often contaminates the groundwater near the main tailing dams, consequently affecting aquatic life [2]. The formation of hazardous cyanide as a byproduct of this process affects both the environment and human health, even in small quantities or low concentration [3]. Cyanide decomposition in tailing dams requires a very long time period [4]. Several methods can be applied to treat the cyanide residue before releasing it to the environment as a chemical waste [5]. Cyanide treatment may include the INCO and activated carbon processes, H_2_O_2_ and ozonation technique, and cyanide recovery by hydrogen. However, the INCO process cannot completely degrade the cyanide species [6]. The activated carbon process can absorb the cyanide species but cannot decompose it [7]. Meanwhile, the H_2_O_2_ oxidation and ozonation technique can treat cyanide, but it requires a very high cost to proceed [8,9]. Lastly, cyanide recovery by hydrogen has been successfully applied to treat cyanide with low-cost consumption, but it does not follow the requirement of the backfilling procedure [10,11].

The membrane technology recently attracted greater attention for extracting metals, non-metal ions, and gaseous and organic compounds, including gold species [12]. This technology, specifically the application of liquid membrane (LM), promises several advantages for the extraction process, including high selectivity and flexibility, resistance to biofouling, and low cost and chemical consumption. An LM consists of four types of membranes, namely bulk LMs (BLMs), emulsion LMs (ELMs), supported LMs (SLMs), and polymer inclusion membranes (PIMs). Unfortunately, BLMs, ELMs, and SLMs have several disadvantages, such as short lifetimes, small interfacial phase areas, and a slow leaching process [13]. PIMs are widely applied in separation and extraction because of their longer lifetime, better stability, and high flexibility [14].

Previous studies have improvised the performance of PIMs by implementing some surface modifications toward these membranes through the incorporation of some additives, including metal azolate framework-4 (MAF-4) [15], titanium oxide [16], aluminum oxide [17], zinc oxide [18], carbon nanotubes [19], and graphene oxide (GO) [20,21]. GO is a low-cost nanomaterial with excellent metal extraction performance. This was reported by a previous study on hexavalent chromium Cr (VI) extraction using a CTA-based membrane with added reduced GO, which revealed 100% of Cr (VI) transport efficiency under optimum conditions and parameters [22]. Moreover, GO is an oxidized form of graphene comprising abundant oxygen-containing groups and forming a metal complex structure by sharing a lone electron pair between GO and metals. The high specific surface area contributed by GO enhances the adsorption capacity for metal extraction [23]. These advantages of GO prompted the current study to integrate GO into PIMs to extract gold from an acidic solution.

## 2. Materials and Methods

### 2.1. Materials

Poly(vinylidenefluoride-co-hexafluoropropylene) (PVDF-co-HFP) (C_5_H_2_F_8_), di-(2-ethylhexyl) phosphoric acid (D_2_EHPA) (C_8_H_17_O)_2_PO_2_H), dioctyl phthalate (DOP) (C_24_H_38_O_4_), tetrahydrofuran (THF) (C_4_H_8_O), and GO (C_14_0H_42_O_20_) were purchased from Sigma Aldrich. The 1000 ppm gold standard solution (Au) in ca.M hydrochloric acid was obtained from Fisher Scientific (Selangor, Malaysia). The sodium sulfite (Na_2_SO_3_) pellets, distilled water, and sodium hydroxide (NaOH) were from Merck (Selangor, Malaysia). Lastly, the 37% hydrochloric acid was from HmbG Chemicals (Hamburg, Germany).

### 2.2. Membrane Preparation

For the membrane preparation, 50 wt.% PVDF-co-HFP as the polymer, 40 wt.% D_2_EHPA as the carrier, and 10 wt.% DOP as the plasticizer were separately weighed in a 50 mL beaker and dissolved in 30 mL THF solvent. The process started from the polymer solution preparation, followed by the addition of the carrier and the plasticizer into the membrane solution with reference to a previous study [24]. A carrier concentration exceeding 50 wt.% is overnice and leads to difficulties in peeling off the membrane from a glass plate. The best composition for the membrane fabrication was 30 to 50 wt.% carrier concentration [25]. GO was added into the membrane mixture with different concentration compositions starting from 0, 0.5, 1.0, 1.5, and 2.0 wt.% labeled as M1, M2, M3, M4, and M5, respectively. Table 1 presents the PIM formulation.

Subsequently, the solution was casted on a glass plate using a casting machine with 0.5 to 2.0 μm thickness. The casted membrane was left to dry under room temperature for 24 h in the fume hood to ensure complete evaporation of the solvent, leaving a transparent and flexible rectangular membrane. The membrane was then cut in half from the glass plate and transferred into an A4 paper.

### 2.3. Membrane Characterization

#### 2.3.1. Scanning Electron Microscopy, Fourier-transform Infrared Spectroscopy, and Thermogravimetric Analyses

JSM-IT100 from JEOL (Tokyo, Japan) was used to analyze the surface morphology of the fabricated GO-PIM membrane. The membrane surface was coated with 5 nm Au–Pt catalyst prior to the scanning electron microscopy (SEM) analysis to make the membranes conductive. The SEM imaging process was performed at 10 kV acceleration voltage. The functional group of the GO-PIM membranes was then determined through Fourier-transform infrared spectroscopy (FTIR) analysis on the fabricated membranes. The Nicolet iZ10 FTIR spectrometer from Thermo Fisher Scientific (Waltham, MA, USA) was used to obtain the FTIR spectra, which had 16 scans with resolutions ranging from 400 to 4000 cm^−1^.

For the thermogravimetric analysis (TGA), the thermogravimetric analyzer from Mettler Toledo (Greifensee, Switzerland) was used to determine the thermal stability of the GO-PIM membrane. The membranes were weighed at 0.002 g each before being placed in a crucible. The TGA experiments were performed in a nitrogen atmosphere at temperatures ranging from 30 °C to 800 °C. The heating rate and the nitrogen gas flow rate were 10 °C min^−1^ and 60 mL min^−1^, respectively.

#### 2.3.2. Ion Exchange Capacity

First, the fabricated membrane was cut into 2 cm × 2 cm pieces and weighed on an analytical balance (ME204E) from Mettler Toledo (Greifensee, Switzerland) before being immersed in 1.0 M HCl for 24 h. The membrane was then removed and thoroughly washed with distilled water to eliminate the excess HCl. Subsequently, the membrane was submerged in 1.0 M NaCl solution for another 24 h, and then removed. The residual solution was titrated using 0.01 M NaOH solution. Phenolphthalein was used as an indicator in the titration. Each titration was performed in triplicate to obtain an accurate result. Equation (2) was used to calculate the membrane’s ion exchange capacity (IEC) [24].

(1) $$\mathrm{IEC}=\frac{ab}{W_{d}}$$

where *a* indicates the concentration of the NaOH solution used for the titration (mol/dm^3^), and *b* is the volume of the NaOH solution (dm^3^). *W_d_* denotes the dry weight of the membrane prior to HCl immersion (g).

#### 2.3.3. Water Uptake

The water uptake analysis was conducted to evaluate the wettability of the fabricated membrane. The membrane was first cut into 2 cm × 2 cm pieces and weighed before a 30 min immersion in distilled water. The membrane was gently dapped with tissue paper after removal from distilled water. Next, the membrane was weighed again to determine the wet weight caused by distilled water absorption. Equation (3) was used to calculate the water uptake of the PIMs [24].

(2) $$\mathrm{Water}\;\mathrm{uptake}=\frac{W_{wet}-W_{dry}}{W_{dry}}\;\times \;100\%$$

where *W_dry_* and *W_wet_* are the dry and wet weights, respectively, of the fabricated membranes before and after distilled water immersion.

### 2.4. Gold Extraction Experiment

**Figure 1:**
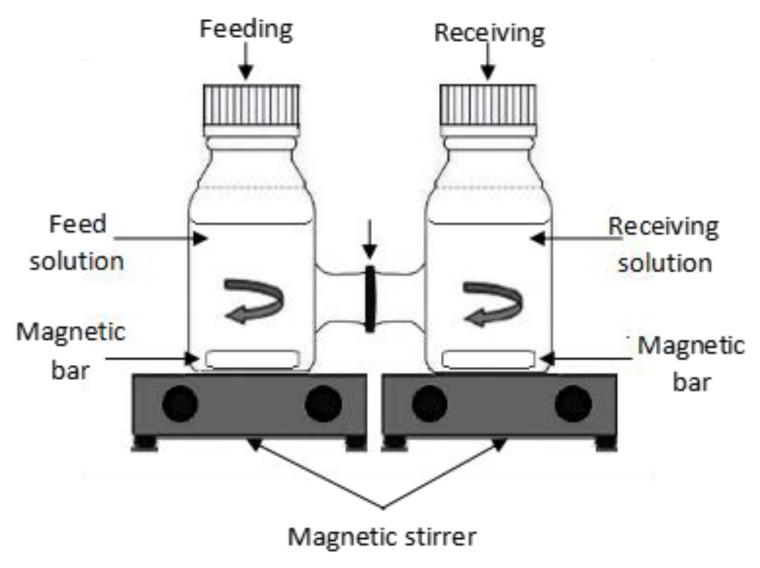
H-cell device used for the diffusion experiment.

The gold extraction experiment was conducted using an H-cell diffusion device composed of two compartments divided by the membrane section. The compartments consisted of feeding and receiving or stripping solutions. Figure 1 depicts the H-cell device structure used for the gold extraction experiment. The feeding solution was filled up with 100 mL of 100 ppm gold standard prepared by diluting 1000 ppm of that gold standard solution in ca.M hydrochloric acid (Fisher Scientific). In addition, 0.100 M sodium sulfite solution (Merck) was prepared by dissolving Na_2_SO_3_ pellets in the distilled water used as the receiving solution [26].

During the diffusion, 5 mL of the sample was taken from the feeding solution at predetermined time intervals (i.e., every 1 h for 5 h periods) to determine the concentration of the gold left in the feeding solution. The Au(III) concentration was determined through atomic absorption spectroscopy (AAS) [26]. The sample was then diluted with hydrochloric acid having a 1:4 (2.5 mL of the sample:7.5 mL HCl) dilution factor before being measured with AAS. The extraction rate percentage (E%) of the GO-PIM membranes for the gold extraction was calculated using Equation (4), where *C_o_* is the initial gold concentration in the aqueous solution (mg/L), and *C_e_* is the final gold concentration in the aqueous solution after the extraction process (mg/L).

(3) $$E\%=\frac{C_{o}-C_{e}}{C_{o}}\;\times \;100$$

## 3. Results and Discussion

### 3.1. Effect of Graphene Oxide on Membrane Characterization

#### 3.1.1. Scanning Electron Microscopy

**Figure 2:**
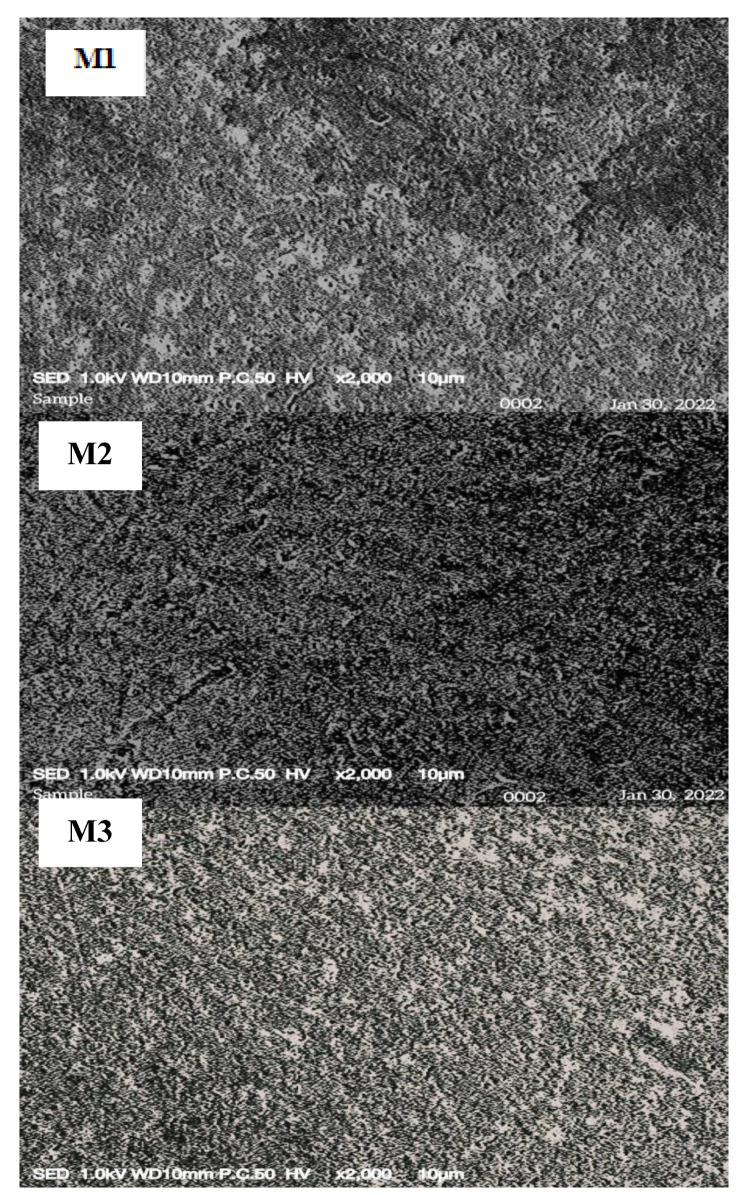

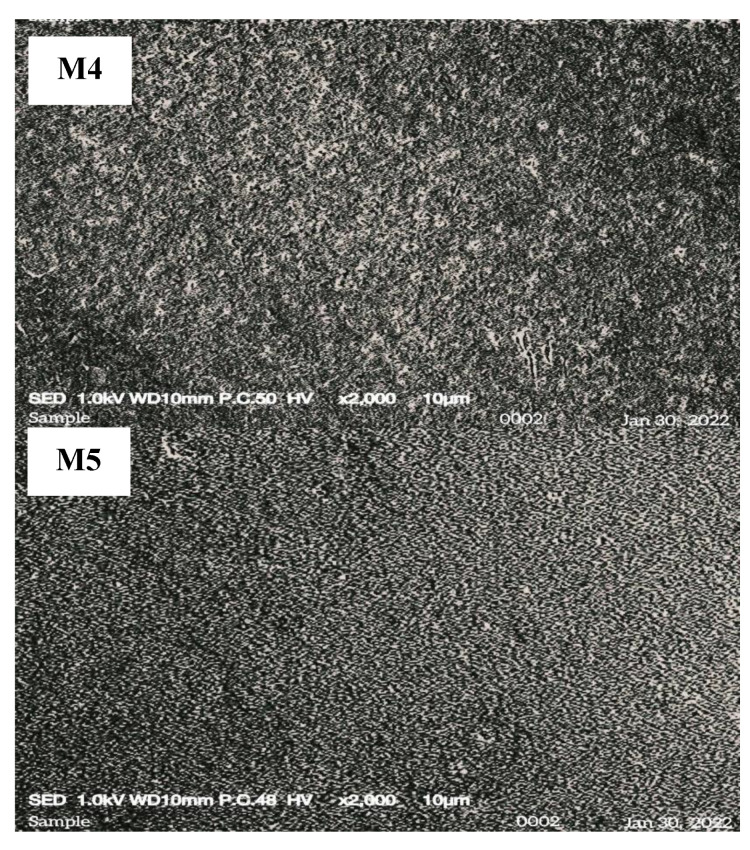
SEM micrographs of M1 to M5 with different GO concentrations.

The surface morphologies of the fabricated membrane were examined through SEM. The membrane structure depends on the method used in the preparation [27]. The solvent evaporation technique using THF was applied to obtain a dense membrane. Figure 2 shows the SEM results of the membrane surfaces. The SEM images of M1 to M5 depict the formation of the membrane porous structure, which indicated that the carrier and the plasticizer in the membrane solution were well distributed within the base polymer [28]. As a function of the presence of the carrier and the plasticizer, the GO-PIM membrane solution became more viscous. This result exhibited the homogeneity of the solution, which led to the ion movement enhancement throughout the membrane caused by the reformation of the membrane micro-structure [29].

Meanwhile, a relatively smooth surface and a dense structure were observed for M2, M3, M4, and M5 after GO addition in the membrane. These results can be attributed to the incorporation of GO into the membrane solution, which led to an increase in the viscosity of the casting solution [30]. The SEM image of M5 shows the smoothest surface with no visible porous structure because it contained the highest GO composition in the membrane. The enhancement of the PIM morphological structure in terms of the membrane surface smoothness was credited to GO addition.

#### 3.1.2. Fourier-Transform Infrared Spectroscopy

**Figure 3:**
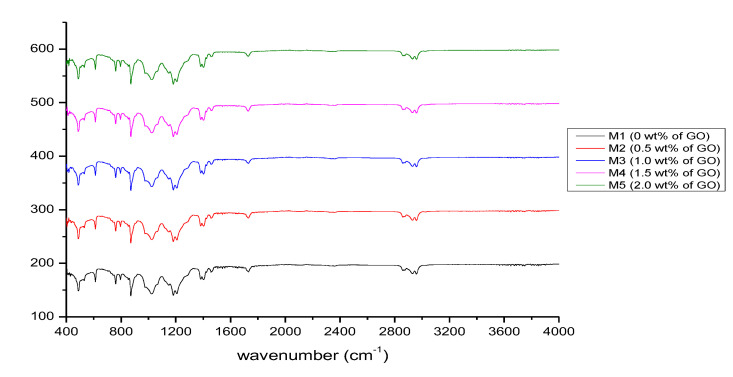
FTIR spectra of the GO-PIM membranes.

The Nicolet iZ10 FTIR spectrometer from Thermo Scientific (Waltham, MA, USA) was used to obtain the FTIR spectra of the membranes. The FTIR data were obtained to determine the functional groups presented in the fabricated membranes. Figure 3 depicts the FTIR spectra of M1 to M5. No significant difference in the bands was identified from the comparison of the peaks of each fabricated PIM. The similar components were determined from the FTIR spectra of the fabricated membranes. The M1 spectra (0.00 wt.% GO) indicated weak peaks of C–H stretching from 2958.59 to 2860.29 cm^−1^ contributed by the C−H bond in D2EHPA and DOP and C=O stretching at 1732.41 cm^−1^. The peaks between 1181.74 and 1149.58 cm^−1^ showed C−F stretching for the fluoro compound of PVDF-co-HFP because the C–F bond is known as the major bond in the PVDF-co-HFP structure.

The peaks at 1403.61 and 1457.57 cm^−1^ exhibited O–H and C–H bending, respectively. The P–O–C and P=O bonds of D2EHPA are depicted by the strong and intense peaks at 1023.06 and 1208.15 cm^−1^, respectively. The bands at 1382.43 and 871.85 cm^−1^ indicated C–O and C–C stretching, respectively. The reduction in the peaks of M2 (1402.99 cm^−1^), M3 (1402.63 cm^−1^), M4 (1402.21 cm^−1^), and M5 (1402.05 cm^−1^) of the O–H bending compared to that of M1 (1403.61 cm^−1^) demonstrated that GO was completely integrated into the polymer matrix of the PVDF-co-HFP-based membrane. This clearly shows that the intermolecular interactions in the membrane are correlated with the PIM compositions. In other words, PIMs have a weak interaction of van der Waals forces or hydrogen bonds between their components [31].

#### 3.1.3. Thermal Gravimetric Analysis

**Figure 4:**
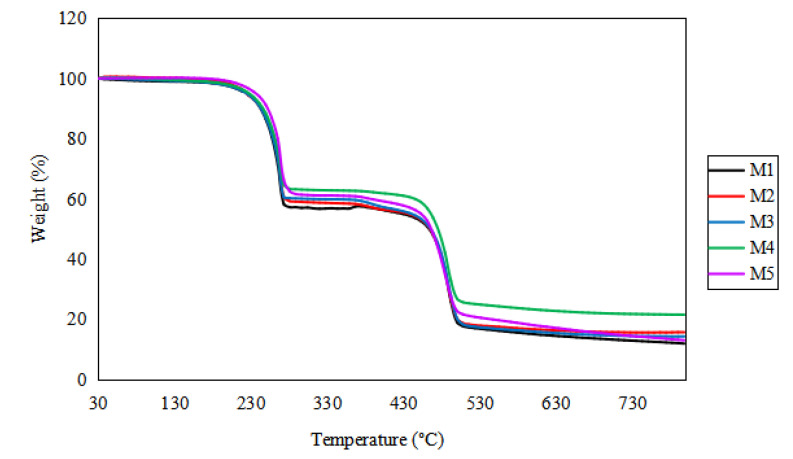
TGA curve for the fabricated GO-PIM membranes.

The thermal gravimetric analysis (TGA) was conducted to measure the thermal stability of a membrane after being exposed to heat under a controlled pressure. The mass of the membrane sample that changed as a function of the temperature revealed its oxidative stability properties and compositional characteristic [32]. Figure 4 presents the curve graph for the TGA analysis. The thermal degradation of the fabricated membranes demonstrated multistep degradation mechanisms. The TGA analysis revealed the significant influence of the presence of GO on the thermal stability of the PIMs. The M1 (0.0 wt.% GO) TGA curve shows a mass loss of 42.79% at 280.67 °C, followed by a weight loss of 52.08% at 468.67 °C. In comparison, the other membranes showed weight losses of 12.61% (M2), 13.04% (M3), 11.55% (M4), and 8.79% (M4) from their initial masses in the major step at temperatures close to 250.00 °C. The thermal stability of GO was reduced at temperatures of approximately 200 °C due to carboxylic degradation and the CO_2_ gas emission [33]. M4 showed the highest thermal stability when compared to M2, M3, and M5, exhibiting a weight loss of 2.16% at 202 °C, 67.33% at 492.17 °C, and 78.54% at 800 °C. The increment of the heat stability of M4 might be attributed to the excellent incorporation of GO in the PVDF-co-HFP polymer. The high thermal decomposition of the membrane is caused by the formation of a strong force between the organic and inorganic phases brought by the addition of GO nanoparticles. Consequently, more heat energy is needed to decompose the membrane chain formation [34].

Despite the fact that M5 composed the highest GO composition, it showed a lower thermal stability compared to M4 because aggregation occurred for 2.0 wt.% GO in the host polymer matrix [35]. The GO-based membranes also outperformed M1 in terms of temperature stability up to 400 °C because GO was successfully incorporated into the PIMs [9]. The GO-based membranes decomposed at a slower rate, implying that heat emission greatly decreased [33].

#### 3.1.4. Ion Exchange Capacity

**Figure 5:**
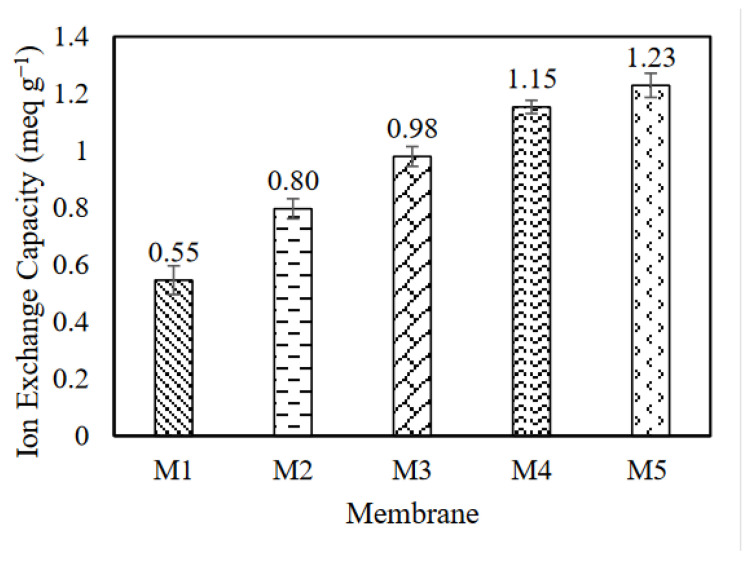
Ion exchange capacity of the fabricated membranes.

The IEC had an ion transportation ability facilitated by the functional groups of the membranes [29]. It was measured to determine the ion site present in the fabricated membranes. Figure 5 illustrates the IEC results for M1 to M5, which reveal an increment of the IEC values with the increasing GO composition. The IEC values increased from 0.55, 0.80, 0.98, 1.15 to 1.23% for M1 (0 wt.% GO), M2 (0.5 wt.% GO), M3 (1.0 wt.% GO), M4 (1.5 wt.% GO), and M5 (2.0 wt.% GO), respectively. The ion conductivity rate was facilitated by the increase of the GO composition in the membrane. The migration of the graphene nanoplates to the membrane surface resulted in the presence of hydroxyl and carboxylic acid groups in the membrane, which enhanced the hydrophilicity and membrane performances [36]. The unique complex of GO after being functionalized (i.e., GO incorporated with polymer membrane) decreased its stacking structure and improved the ion transport efficiency [37]. The hydrophilicity of the membrane also increased significantly because the polar element on the surface free energy increased [38].

#### 3.1.5. Water Uptake

**Figure 6:**
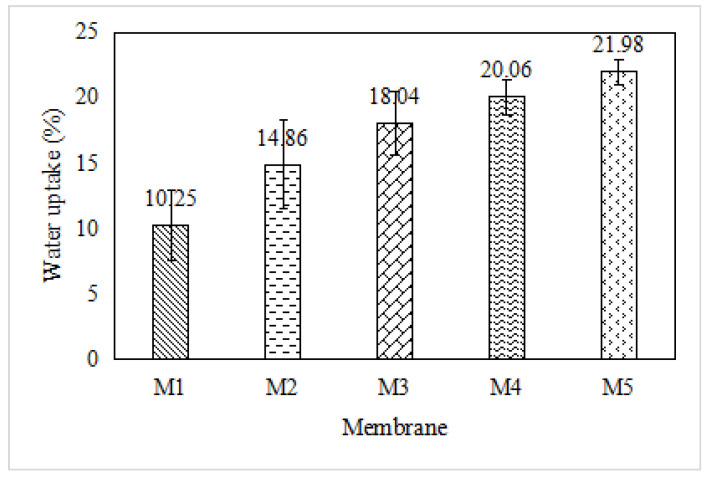
Water uptake of the fabricated GO-PIM membranes.

Water uptake would commonly be matched up with the IEC [39]. A high IEC will lead to a high water uptake, which will then result in an increment of the water permeability [39]. Figure 6 displays the water uptake of the fabricated PIMs with different GO concentrations. The results of the water uptake analysis of the PIMs indicate that the membrane hydrophilicity is influenced by the GO addition. The M1 PIM (0.0 wt.% GO) showed the lowest water uptake percentage of 10.25%. In contrast, the M5 PIM revealed the highest water uptake value of 21.98%. The water uptake percentage depicted an overall increase with an increase in the GO composition in the membrane, as explained by the hydrophilicity of the PIMs enhanced by the GO addition in the range of 0.5 to 2.0 wt.%. Theoretically, the hydrophobic nature repels water, leading to a decrement in the water permeability. By contrast, the hydrophilic nature depicts the tendency of adsorbing water [40]. The hydrophilic nature proposed by GO improved the water permeability of the membranes by attracting the water molecules inside the membrane matrix and providing additional pathways for the water molecules to pass through the membrane [41].

The water uptake and the IEC of the membranes were mutually related. The water uptakes were dependent on the chemical stability and the ion conductivity of the membranes [42].

### 3.2. Effect of the GO Content on the Membrane Performance

**Figure 7:**
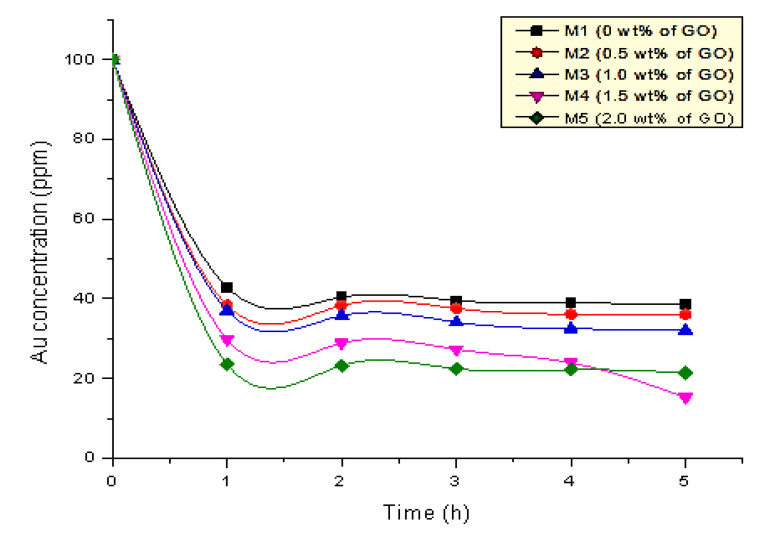
Curve graph of the gold extraction for the five GO-PIM membranes.

The extraction of 100 ppm gold (III) from hydrochloric acid solution was studied herein using the H-cell apparatus consisting of fabricated PIMs. The diffusion of gold from the feeding solution into the membrane involved the passing through of gold to the aqueous stagnant layer of the feeding solution, followed by the diffusion of gold into the membrane phase [1]. In this work, membranes containing 0, 0.5, 1.0, 1.5, and 2.0 wt.% GO were used. Figure 7 presents the extraction rate results.

The results showed that the gold (III) concentration in the feeding solution decreased within time. The ability of the PIMs to extract gold was attributed to the presence of an extractant (carrier) (i.e., D_2_EHPA in this study). This liquid complexing agent was responsible for binding and transporting the selected species that flowed out across the PIMs [26]. The adsorption mechanism of this process are electrostatic interaction and hydrogen bonding brought by the carboxylate ions and hydroxyl groups with the gold metals. The same mechanism was shown by previous study in which the adsorption of phosphate from wastewater via lanthanum carbonate grafted ZSM-5 adsorbent (LC-ZSM-5) through the exchanged of lanthanum carbonate (LC) to lanthanum phosphate (LP) [43]. The GO addition to the PIMS also increased the membrane efficiency for gold extraction. M1 exhibited the lowest Au extracted because this membrane was fabricated without a GO composition.

As mentioned, adding GO enhances the membrane efficiency for gold extraction. The GO incorporated membranes (i.e., M2 to M5) showed higher extraction rates than M1 without a GO concentration. The incorporation of graphene nanoparticles into the polymeric membranes resulted in a remarkable reinforcement on the membrane performances in terms of thermal stabilities [44], morphologies, and structural properties [36]. In this case, the high gold extraction rate shown by the GO-based membrane was caused by the improvement of the membrane porosity and led to the increment of the ionic conductivity [35].

The similar results were reported by Kaya et al. (2016), whose study showed an improvement in the membrane properties when reduced GO was added into a CTA polymer membrane for hexavalent chromium Cr (VI) extraction and recovery [15]. Gold recovery using graphene oxide/calcium alginate hydrogel beads from a detector was also studied. The maximum Au uptake was more than 95% and highly depended on the solution’s pH. An excellent extraction performance was observed for the 2–4 pH range in acidic media [37]. In short, applying GO improved the extraction efficiency with optimum parameter.

Figure 7 shows the variance on the extraction rate for M2, M3, M4, and M5 with different GO compositions. M4 with 1.5 wt.% GO yielded the best extraction rate of 84.71%. During the diffusion experiment on M4, the feed solution turned from a gold yellow color to a colorless state. This phenomenon might have happened because of the high ionic conductivity provided by GO with 1.5 wt.% composition. Meanwhile, the PIMs with GO concentrations above 1.5 wt.% (M5) were not that efficient in gold extraction because of the filler aggregation at a higher composition, which resulted in the ionic conductivity reduction [35].

## 4. Conclusions

In this work, PIMs were prepared using PVDF-co-HFP as the base polymer, D_2_EHPA as the carrier, and DOP as the plasticizer and by adding GO with different compositions (i.e., within 0 to 2.0 wt.%) in the casting solution. The characterization results showed enhancement in terms of the morphological structure, water uptake, ion exchange capacity, and thermal stability of the fabricated membranes in the presence of GO. In the addition of GO, membrane morphologies revealed a very smooth structure indicating the incorporation of GO into the membrane. Reduction O-H bending peaks of FTIR spectra proposes that the hydroxyl group attributed by GO was involved in the adsorption of gold metal process. M4 with 1.5 wt.% of GO composition exhibited efficient gold extraction performance with 84.71% extraction. This result is attributed to the excellent incorporation of 1.5 wt.% of GO into PVDF-co-HFP. The GO composition exceeding 1.5 wt.% showed a decrement on the membrane efficiency toward gold extraction because of the agglomeration of the GO particles with the host polymer, which affected the membrane performance.

## Figures and Tables

**Table 1:** Membrane solution formulation with different GO compositions.

Membrane	Polymer (wt.%)	Carrier (wt.%)	Plasticizer (wt.%)	Nanoparticles (wt.%)
	PVDF-co-HFP	D_2_EHPA	DOP	GO
M1	50.0	40.0	10.0	0.0
M2	50.0	40.0	10.0	0.5
M3	50.0	40.0	10.0	1.0
M4	50.0	40.0	10.0	1.5
M5	50.0	40.0	10.0	2.0

## Data Availability

The data presented in this study are available within the present article.

## References

[1] Wang Z., Sun Y., Tang N., Miao C., Wang Y., Tang L., Wang S., Yang X. (2019). Simultaneous extraction and recovery of gold(I) from alkaline solutions using an environmentally benign polymer inclusion membrane with ionic liquid as the carrier. Sep. Purif. Technol..

[2] Cho Y., Cattrall R.W., Kolev S.D. (2017). A novel polymer inclusion membrane based method for continuous clean-up of thiocyanate from gold mine tailings water. J. Hazard. Mater.

[3] Hou D., Liu L., Yang Q., Zhang B., Qiu H., Ruan S., Chen Y., Li H. (2020). Decomposition of Cyanide from Gold Leaching Tailings by Using Sodium Metabisulphite and Hydrogen Peroxide. Adv. Mater. Sci. Eng..

[4] Ebbs S. (2004). Biological degradation of cyanide compounds. Curr. Opin. Biotechnol..

[5] Razanamahandry L.C., Onwordi C.T., Saban W., Bashir A.K.H., Mekuto L., Malenga E. (2019). Performance of various cyanide degrading bacteria on the biodegradation of free cyanide in water. J. Hazard. Mater..

[6] Breuer P.L., Hewitt D.M. (2019). INCO Cyanide destruction insights from plant reviews and laboratory evaluations. Miner. Process. Extr. Metall.

[7] Jauto A.H., Memon S.A., Channa A., Hussain A. (2019). Environmental Effects Efficient removal of cyanide from industrial effluent using acid treated modified surface activated carbon. Energy Sources, Part A Recover. Util. Environ. Eff..

[8] Pueyo N., Miguel N., Ovelleiro J.L., Ormad M.P. (2016). Limitations of the removal of cyanide from coking wastewater by ozonation and by the hydrogen peroxideozone process. Water Sci. Technol..

[9] Yang T., Cao J., Cao X., Dong Z., Yang Z., Chen Z., Qiu S. (2020). Experimental study on cyanide-contaminated soil (China) treatment by leaching and decomposition. Environ. Sci. Pollut. Res..

[10] Yazıcı E.Y., Deveci H., Yılmaz E., Ahlatcı F., Celep O. (2017). Recovery of Cyanide from Effluents Using Carbon Dioxide. Mugla. J. Sci. Technol..

[11] Parga J.R., Valenzuela J.L., Moreno H., Pérez J.E. (2011). Copper and Cyanide Recovery in Cyanidation Effluents. Adv. Chem. Eng. Sci..

[12] Bhattacharya M., Mandal M.K. (2017). Synthesis and characterization of ionic liquid based mixed matrix membrane for acid gas separation. J. Clean. Prod..

[13] Parhi P.K. (2013). Supported liquid membrane principle and its practices: A short review. J. Chem..

[14] Li H., Wang X., Cao L., Zhang X., Yang C. (2015). Gold-recovery PVDF membrane functionalized with thiosemicarbazide. Chem. Eng. J..

[15] Wu R., Tan Y., Meng F., Zhang Y., Huang Y. (2022). PVDF/MAF-4 composite membrane for high flux and scaling-resistant membrane distillation. Desalination.

[16] Bet-Moushoul E., Mansourpanah Y., Farhadi K., Tabatabaei M. (2016). TiO2 nanocomposite based polymeric membranes: A review on performance improvement for various applications in chemical engineering processes. Chem. Eng. J..

[17] Garcia-Ivars J., Iborra-Clar M.I., Alcaina-Miranda M.I., Mendoza-Roca J.A., Pastor-Alcañiz L. (2016). Surface photomodification of flat-sheet PES membranes with improved antifouling properties by varying UV irradiation time and additive solution pH. Chem. Eng. J..

[18] Tan Y.H., Goh P.S., Ismail A.F., Ng B.C., Lai G.S. (2017). Decolourization of aerobically treated palm oil mill effluent (AT-POME) using polyvinylidene fluoride (PVDF) ultrafiltration membrane incorporated with coupled zinc-iron oxide nanoparticles. Chem. Eng. J..

[19] Tijing L.D., Woo Y.C., Wang-Geun S., Tao H., June-Seok C., Seung-Hyun K., Ho K.S. (2016). Superhydrophobic nanofiber membrane containing carbon nanotubes for high-performance direct contact membrane distillation. J. Memb. Sci..

[20] Pandey R.P., Shukla G., Manohar M., Shahi V.K. (2017). Graphene oxide based nanohybrid proton exchange membranes for fuel cell applications: An overview. Adv. Colloid Interface Sci..

[21] Chen Y., Long J., Xie B., Kuang Y., Chen X., Hou M., Gao J., Liu H., He Y., Long C. (2022). One-Step Ultraviolet Laser-Induced Fluorine-Doped Graphene Achieving Superhydrophobic Properties and Its Application in Deicing. ACS Appl. Mater. Interfaces.

[22] Kaya A., Canan O., Korkmaz H.A., Shilpi A., Vinod K.G., Necip A., Aydan Y. (2016). Reduced graphene oxide based a novel polymer inclusion membrane: Transport studies of Cr (VI). J. Mol. Liq..

[23] Liu L., Luo X.B., Ding L., Luo S.L., Luo X., Deng F. (2018). Application of Nanotechnology in the Removal of Heavy Metal from Water. Nanomaterials for the Removal of Pollutants and Resource Reutilization.

[24] Soo J.A.L., Shoparwe N.F., Otitoju T.A., Mohamad M., Tan L.S., Li S., Makhtar M.M.Z. (2021). Characterization and kinetic studies of poly(Vinylidene fluoride-co-Characterization and kinetic studies of poly(Vinylidene fluoride-co-hexafluoropropylene) polymer inclusion membrane for the malachite green extraction. Membranes.

[25] Abdul-halim N., Whitten P.G., Nghiem L.D. (2013). Characterising poly (vinyl chloride)/Aliquat 336 polymer inclusion membranes: Evidence of phase separation and its role in metal extraction. Sep. Purif. Technol..

[26] Bonggotgetsakul Y.Y.N., Cattrall R.W., Kolev S.D. (2015). Extraction of gold (III) from hydrochloric acid solutions with a PVC-based polymer inclusion membrane (PIM) containing cyphos® IL 104. Membranes.

[27] Sellami F., Kebiche-senhadji O., Marais S., Colasse L. (2019). Separation and Puri fi cation Technology Enhanced removal of Cr (VI) by polymer inclusion membrane based on poly (vinylidene fl uoride) and Aliquat 336. Sep. Purif. Technol..

[28] Caprarescu S., Miron A.R., Purcar V., Radu A.L., Sarbu A., Nicolae C.A., Pascu M., Ion-Ebrasu D., Raditoiu V. (2018). Treatment of Crystal Violet from Synthetic Solution Using Membranes Doped with Natural Fruit Extract. Clean Soil Air Water.

[29] Dharmalingam S., Kugarajah V., Mohan S.V. (2019). Biomass, Biofuels and Biochemicals, Microbial Electrochemical Technology.

[30] Haiyang Z., Liguang W., Zhijun Z., Lin Z., Huanlin C. (2013). Improving the antifouling property of polysulfone ultrafiltration membrane by incorporation of isocyanate-treated graphene oxide. Phys. Chem..

[31] Mohd Amin N.H., Mehamod F.S., Mohd Suah F.B. (2019). A novel approach in simultaneous extraction of basic dyes by using a batch reactor consisting a polymer inclusion membrane. Alex. Eng. J..

[32] Ling Y.Y., Mohd Suah F.B. (2017). Extraction of malachite green from wastewater by using polymer inclusion membrane. J. Environ. Chem. Eng..

[33] Najafi F., Rajabi M. (2015). Thermal gravity analysis for the study of stability of graphene oxide–glycine nanocomposites. Int. Nano Lett..

[34] Xiaorui Z., Xue S., Tong L., Ling W., Minghe C., Jiahao S., Siqi Z. (2020). Preparation of PI porous fiber membrane for recovering oil-paper insulation structure. J. Mater. Sci. Mater. Electron..

[35] Ahmad A.L., Farooqui U.R., Hamid N.A. (2018). Effect of graphene oxide (GO) on Poly(vinylidene fluoride-hexafluoropropylene) (PVDF- HFP) polymer electrolyte membrane. Polymer.

[36] Zinadini S., Akbar A., Rahimi M., Vatanpour V. (2014). Preparation of a novel antifouling mixed matrix PES membrane by embedding graphene oxide nanoplates. J. Memb. Sci..

[37] Long C., Lu C., Li Y., Wang Z., Zhu H. (2020). N-spirocyclic ammonium-functionalized graphene oxide-based anion exchange membrane for fuel cells. Int. J. Hydrogen Energy..

[38] Yuhai L., Bai Q., Guan Y., Zhang P., Shen R., Li L., Liu H., Yuan X., Miao X., Han W. (2022). In situ plasma cleaning of large-aperture optical components in ICF. Nucl. Fusion..

[39] Luque J., Salvo D., de Luca G., Cipollina A., Micale G. (2020). Effect of ion exchange capacity and water uptake on hydroxide transport in PSU-TMA membranes: A DFT and molecular dynamics study. J. Memb. Sci..

[40] Arkles B. (2006). Hydrophobicity, hydrophilicity and silanes. Paint. Coat. Ind..

[41] Bano S., Mahmood A., Kim S., Lee K. (2014). Membrane with improved flux and antifouling properties. J. Mater. Chem. A.

[42] Msomi P.F., Nonjola P.T., Ndungu P.G., Ramontja J. (2020). ScienceDirect anion exchange membrane blended with TiO 2 with improved water uptake for alkaline fuel cell application. Int. J. Hydrog. Energy.

[43] Yang Y., Wang Y., Zheng C., Lin H., Xu R., Zhu H., Bao L., Xu X. (2022). Lanthanum carbonate grafted ZSM-5 for superior phosphate uptake: Investigation of the growth and adsorption mechanism. Chem. Eng. J..

[44] Terrones M., Martín O., González M., Pozuelo J., Serrano B., Cabanelas J.C., Vega-díaz S.M., Baselga J. (2011). Interphases in graphene polymer-based nanocomposites: Achievements and Challenges. Adv. Mater..

